# Incidence, morbidity and mortality of admissions related to alcohol consumption on critical care: a single-centre experience

**DOI:** 10.1186/cc10999

**Published:** 2012-03-20

**Authors:** A Retter, F Tait, M Stockwell

**Affiliations:** 1 St Helier Hospital, London, UK

## Introduction

Excessive alcohol consumption is a major challenge to public health. In 2000 it accounted for 4% of the global disease burden. However, the relationship between alcohol and health is complex and the burden it places on admissions to critical care is uncertain.

## Methods

We conducted a retrospective analysis of prospectively collected data on the influence of excess alcohol consumption on the outcome of patients admitted from July 2009 to July 2011. The admitting physician determined the relationship between alcohol use and admission. No patients were excluded. All continuous data are expressed as medians and were compared using the Wilcoxon Mann-Whitney U test. Categorical data were compared using the chi-squared test.

## Results

A total of 1,150 patients were admitted, 129 cases (11.2%) were identified as having excess alcohol consumption. Of these cases 34% were women, whilst 48% of the controls were female. The median age of the cases was 54 years versus 68 years for the controls (*P *< 0.001). The cases had a lower APACHE II score, 14.3 vs. 15.8 (*P *= 0.002). Twenty-four (18.6%) of the cases with excess alcohol consumption died on the ICU compared to 141 controls (13.8%) (*P *> 0.1). The hospital mortality was similar between the two groups, 28 (21.7%) against 215 (21.1%) controls (*P *> 0.5). The cases spent longer on the ICU, median 3.95 days versus 2.9 in the controls (*P *< 0.001). On admission the cases required a median of 2.0 organ supportive therapies compared to 1.8 in the control group (*P *< 0.001). The cases were ventilated for a mean of 4.1 days compared to 2.4 days in the controls (*P *< 0.001). There was no difference in the rate of sepsis between either group, 10% in the cases and 9.8% in the controls. Twenty-six patients were admitted with known alcoholic cirrhosis (0.23%), 10 with oesophageal varices and three with acute pancreatitis related to alcohol.

## Conclusion

To our knowledge this is the largest single-centre assessment of the burden of excess alcohol consumption on patients admitted to critical care. Eleven per cent of all admissions to the ICU were complicated by excess alcohol consumption. The ITU mortality of these patients was increased when compared to the controls, despite the patients having an equivalent APACHE score on admission and tending to be younger. The cases spent less time in hospital than the controls. This was due to a bimodal distribution of their survival curve. Our study is limited by its retrospective design and the risk of selection bias.

